# Species diversity and distribution of freshwater molluscs of Javakheti Highlands (Republic of Georgia)

**DOI:** 10.3897/BDJ.9.e66649

**Published:** 2021-06-07

**Authors:** Ani Bikashvili, Nino Kachlishvili, Levan Mumladze

**Affiliations:** 1 Institute of Zoology, Ilia State University, Tbilisi, Georgia Institute of Zoology, Ilia State University Tbilisi Georgia

**Keywords:** freshwater mollusca, Javakheti Highlands, Georgia, diversity

## Abstract

The diversity and distribution of freshwater molluscs is poorly studied in the Republic of Georgia, due to the scarcity of field studies during the last 50 years. Here, we present the results of the first concerted investigation of freshwater mollusc biodiversity in the Javakheti Highlands, in the southern, mountainous region of Georgia. In total, we were able to collect 22 species from 42 sampling localities, including different kinds of freshwater habitats. Amongst the 22 collected species, 12 were recorded for the first time from Javakheti. From the newly-recorded species, *Bathyomphalus contortus* is a new country record, whose identity is supported by 16S rRNA sequence data.

## Introduction

Freshwater wetlands, while providing irreplaceable ecosystem services (Aylward et al. 2005, Carpenter et al. 2011), are, on the other hand, the most threatened ecosystems worldwide (Dudgeon 2019). Due to ever increasing demand on freshwater resources and other direct or indirect anthropogenic influences, the conservation of freshwater biodiversity and maintenance of freshwater ecosystem function remain a significant challenge (Dudgeon et al. 2006). This concern is particularly acute in undeveloped parts of the world, where the knowledge of freshwater biodiversity remains scarce. As an example, understanding of freshwater biodiversity of the Republic of Georgia, this being the central part of the Caucasus biodiversity hotspot (Mittermeier et al. 2011, Myers et al. 2000), is scanty and outdated (Japoshvili et al. 2016, Mumladze et al. 2020). This makes it impossible to assess freshwater ecosystem conservation priorities at the national and international scale (Mumladze et al. 2020). Similar to many other taxa, Georgian freshwater molluscs are poorly studied, both taxonomically and ecologically. The first and last summarised check-list, providing data on the distribution of 59 freshwater mollusc species, was published 47 years ago (Javelidze 1973). This old source includes fragmented data on freshwater molluscan taxa collected during or prior to the Soviet period. Unfortunately, no significant progress has been made since then. Indeed, only a few research papers have been published since, reporting either new species descriptions or new invasive records (Vinarski et al. 2014, Vinarski and Palatov 2018, Mumladze et al. 2019a, Mumladze et al. 2019b, Chertoprud et al. 2020, Grego et al. 2020, Neiber et al. 2021). Perhaps the most remarkable study was published in 2020 by Grego and co-authors, when 21 new freshwater subterranean species were described from a small region of central-western Georgia. However, interest in the diversity of Georgian freshwater molluscs has gained new momentum in the last decade, though the available data are still very fragmented and incomplete. The aim of the present manuscript is to describe the results of the first inventory of freshwater mollusc diversity of the southern mountainous region of Georgia (Javakheti Highlands), which includes some new faunistic records for the Region and a new country record of the species *Bathyomphalus contortus* Linnaeus, 1758.

## Materials and Methods

### Study area

**Figure 1.**
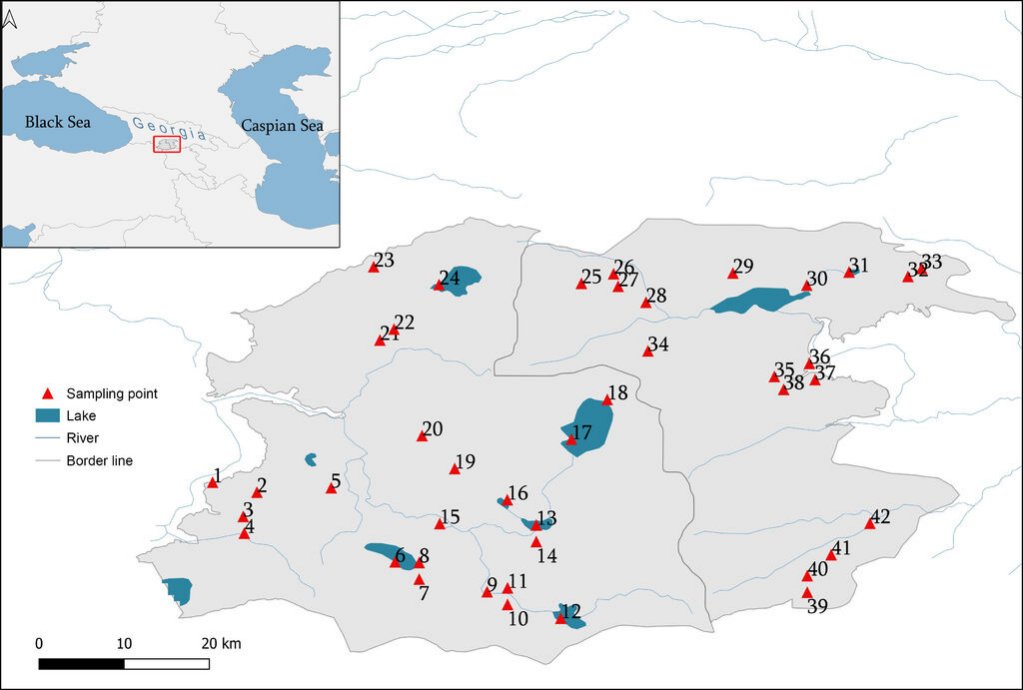
Map of the sampling area and sampling sites. The locality numbers correspond to the descriptions given in Table 1.

The Javakheti Highlands are located in southern Georgia, in the central part of the Lesser Caucasus Mountains (Fig. 1). The Highlands are of entirely volcanic origin, composed of basaltic – andesitic lavas, with an altitudinal range between 1200 – 3300 m a.s.l., Didi Abuli (3300 m a.s.l.) being the highest peak. There are two mountain ranges in Javakheti – the Abul-Samsari and Javakheti ranges, both running from north to south. Though all the area drains into the Kura River, the basin is divided into two parts by the Abul-Samsari and Javakheti ranges: 1) the western Javakheti Plateau (elevation profile ranging from 1700 to 3300 m a.s.l.) drained by the Paravani River and 2) the eastern Tsalka Plateau (elevation profile - from 1200 to 2800 m a.s.l.), part of the Khrami River basin (Adamia et al. 2011, Paffenholz 1963, Tielidze 2019). There are ca. 250 km between the confluences of these rivers with the Kura River. These Highlands are amongst the most important wetland areas in the region, due to the wealth of freshwater sources. More than 60 lakes, various types of wetlands (except peat bogs) and rivers are present. Terrestrial habitats of this Region include mountain steppes (with different land-use regimes) and the Region is essentially devoid of forest, with 0.1% natural and 0.3% of artificial (pine) forest cover. The Region is characterised by a continental climate, with an average annual precipitation of 500 – 700 mm and an average annual temperature of 3 - 5°C (Matcharashvili et al. 2004, Maruashvili 1964).

### Data collection

Sampling of freshwater molluscs was carried out within the period of 2015 – 2019 from 42 sampling sites (Table 1), including a variety of freshwater habitats including running and stagnant waters and swamps. Mollusc specimens were collected using kick – netting and also hand collecting during each sampling event. Although our sampling strategy was not strictly quantitative, we tried to exhaustively sample each site. Doing so, we took a number of samples (minimum three sub-samples in small stagnant waters or river sites, up to 12 widely-separated sub-samples for larger lakes) from each of 42 sites, in order to cover all the microhabitats as effectively as possible. Where only one or two specimens of any one species were collected, repeated sampling in subsequent years was performed. Collected specimens were immediately fixed in 96% ethanol and later identified to a species level. Keys of Glöer (2019), Glöer (2002), Piechocki and Wawrzyniak-Wydrowska (2016), Piechocki (1989) and Vinarski et al. (2020) were used for species identification. Specimen identification were mainly based on shell shapes supplemented by anatomical genetic studies where possible. Voucher materials were deposited in the collection of the Institute of Zoology of Ilia State University (Tbilisi).

### Data analyses

Sampling completeness for freshwater molluscs of Javakheti Highlands was checked by sample-based rarefaction analyses, using presence/absence data. This technique gives an overview of uncertainty of total species richness for the given region, related to incomplete sampling (Chao et al. 2014). The calculation was performed using the ‘iNEXT’ R package (R Core Team 2020, Hsieh et al. 2016). Shells were measured using digital calipers with 0.01 mm accuracy. Standard measurements of shell and aperture height and width of each collected taxon were made. We also measured seven characters according to methods described in Soldatenko and Starobogatov (2004) for 39 specimens of the genus *Ancylus* from various sampling locations, in order to quantify the apparent shell-shape variation. In particular, the measurements of shell length (SL), shell width (SW), shell height (SH), shortest distance between apex and apertural margin (Ra), shortest distance between highest point and apertural margin (Rh) and height of lowered part of shell near apex (Hsp) were recorded. The measurements (Suppl. material 1) were then subjected to unsupervised multivariate ordination for Principal Component Analyses (PCA), in order to reveal and visualise multivariate differentiation between the forms, using the R packages ‘factoextra’ and ‘FactoMineR’ (Lê et al. 2008, Kassambara 2017, Kassambara and Mundt 2020).

We also subjected part of the samples to DNA barcoding to check the validity of morphological identifications. We extracted genomic DNA from foot tissue, using DNeasy Blood & Tissue Kits according to manufacturer instructions (DNeasy Blood & Tissue 2020). A mitochondrial gene fragment of the 16S ribosomal RNA subunit (16S rRNA) was amplified using the primers 16SF (forward) 16SR (reverse) of Palumbi et al. 1991). Conditions for Polymerase Chain Reaction (PCR) were adopted from Wethington and Lydeard (2007) (94°C 3 min, (94°C 40 s, 48°C 60 s, 72°C 60 s) × 30, 72°C 10 min). 16S rRNA amplicons were sequenced at Macrogen Europe Laboratory (Amsterdam, Netherlands). In addition, cytochrome oxidase c subunits I (COI) were amplified using Folmer et al. (1994) forward (LCO1490COI) and Kuhn’s reverse (LCO1491) primers (cited in Cordellier and Pfenninger (2008)). The PCR conditions were employed from Cordellier and Pfenninger (2008) (92°C 2 min, (92°C 40 s, 40-52°C 60 s, 68°C 90 s) × 35, 68°C 90 s). Sequencing reactions were performed using Big Dye Terminator v.3.1 (Applied Biosystems, Foster City, CA, USA) and were sequenced on an automated sequencer. Sequences were checked against the NCBI database, using the BLASTn search (Altschul et al. 1990). Kimura-2-parameter distances were calculated and Neighbour – Joining (NJ) trees were constructed using MEGA X software (Kumar et al. 2018).

## Results

Our study found 22 freshwater mollusc species belonging to 15 genera and four families recorded from 42 sites. The list of species and their distributions in the Javakheti Highlands are shown in Table 2 and the supplementary figures (Suppl. material 2). The most species-rich family were Planorbidae with 10 species, followed by Lymnaeidae (6 species), Sphaeriidae (6 species) and Physidae (1 species).

**Figure 2.**
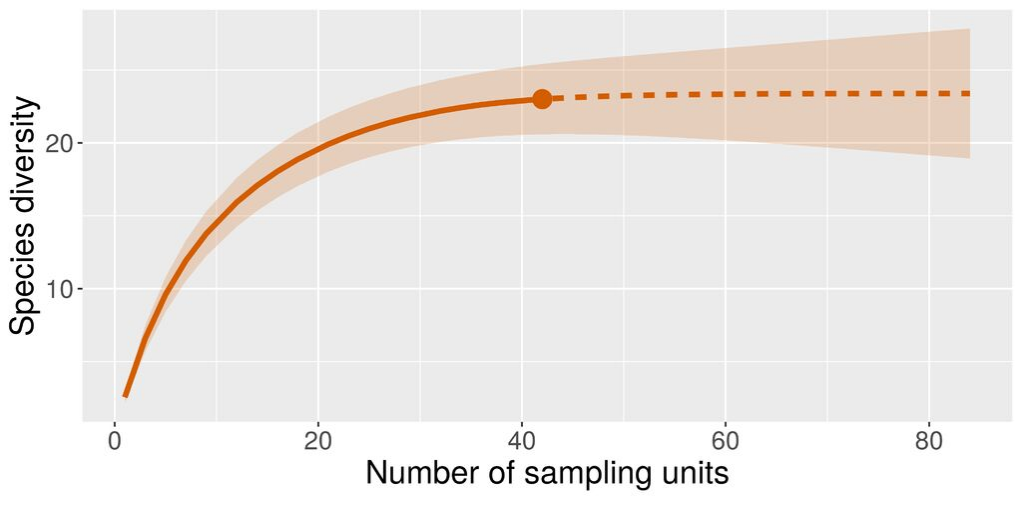
Sample-based rarefaction of freshwater mollusc inventory data from Javakheti Highlands, based on 22 species from 42 sampling localities. Dashed part of the curve indicates the extrapolation while the shaded area indicates the confidence intervals of the rarefaction curve.

Overall, the western part of Javakheti Highlands (basin of River Paravani), with 19 species of freshwater molluscs, was richer compared to the eastern part (basin of River Khrami) with 15 species. However, the studied freshwater bodies are a just tiny part of the total freshwater habitats in the Region. Thus, the obtained spatial distribution of each species must be considered as preliminary. On the other hand, the total, regional species count should be considered nearly complete. Indeed, according to sample-based rarefaction, on average, no additional species is expected after doubling the sampling effort (with upper confidence limit of 27 species) (Fig. 2).

**Figure 3.**
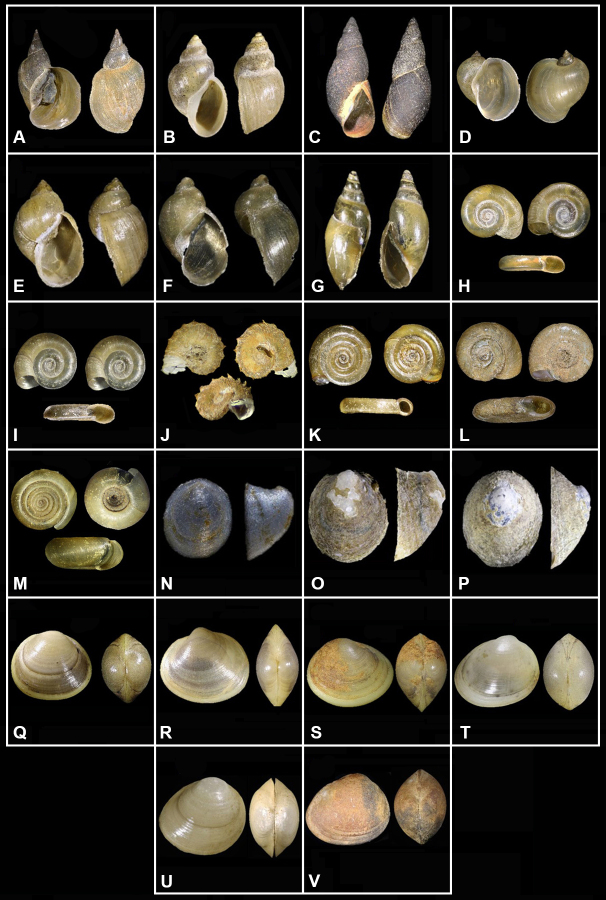
Shells of the freshwater mollusc species collected in Javakheti Highlands during this study. **A.** *Lymnaea stagnalis*, H – 19.5 mm, W - 10 mm; **B.** *Galba truncatula*, H - 5 mm, W – 3.4 mm; **C.** *Stagnicola palustris*, H - 12 mm, W – 4.5 mm; **D.** *Radix auricularia*, H – 15.8 mm, W – 10.6 mm; **E.** *P. peregra*, H - 10.5 mm, W – 5.8 mm; **F.** *Ampullaceana lagotis*, H – 8.3 mm, W - 5 mm; **G.** *Aplexa hypnorum*, H – 5.3 mm, W – 3 mm; **H.** *Planorbis planorbis*, H – 2.5 mm, W – 15 mm; **I.** *P. intermixtus*, H – 1.3 mm, W – 7.5 mm; **J.** *Armiger crista*, H – 0.6 mm, W – 2.8 mm; **K.** *Anisus leucostoma*, H – 1.4 mm, W – 6.3 mm; **L.** *A. spirorbis*, H – 1.4 mm, W – 4.6 mm; **M.** *Bathyomphalus contortus*, H – 1.6 mm, W – 5.3 mm; **N.** *Ancylus major*, L – 5.6 mm, H – 2.6 mm. W – 4.4 mm; **O.** *A. benoitianus*, L – 5.4 mm, H – 2.6 mm, W – 4.3 mm; **P.** *Ancylus* sp., L – 4.4 mm, H – 1.3 mm, W – 3.8 mm; **Q.** *Sphaerium corneum*, L – 10 mm, H -7.5 mm, W – 7.6 mm; **R.** *Musculium lacustre*, L – 7.8 mm, H – 6.5 mm, W – 4.5 mm; **S.** *Pisidium casertana*, L – 4.6 mm, H – 3.5 mm, W – 2.5 mm; **T.** *P. subtruncata*, L – 3.8 mm, H – 3 mm, W – 2.8 mm; **U.** *P. nitidum*, L – 3.3 mm, H – 2.8 mm, W – 2.2 mm; **V.** *P. obtusale*, L – 3 mm, H – 2.6 mm, W – 2 mm.

Amongst the collected species, we detected all seven species previously known from the region (Table 2, Fig. 3). In addition, six planorbid (Ancylus cf. benoitianus, A. cf. major, *Planorbis intermixtus*, *Anysus leucostoma*, *Anisus spirorbis*, *Bathyomphalus contortus*) and, as yet, unidentified form of *Ancylus*, one lymnaeid (*Stagnicola palustris*), one physid *(Aplexa hypnorum*) and four sphaeriid species (*Sphaerium corneum*, *Musculium lacustre*, *Pisidium nitidum*, *Pisidium obtusale*) were firstly recorded for the Javakheti Highlands, while *Bathyomphalus contortus* is a new country record for Georgia.

**Figure 4.**
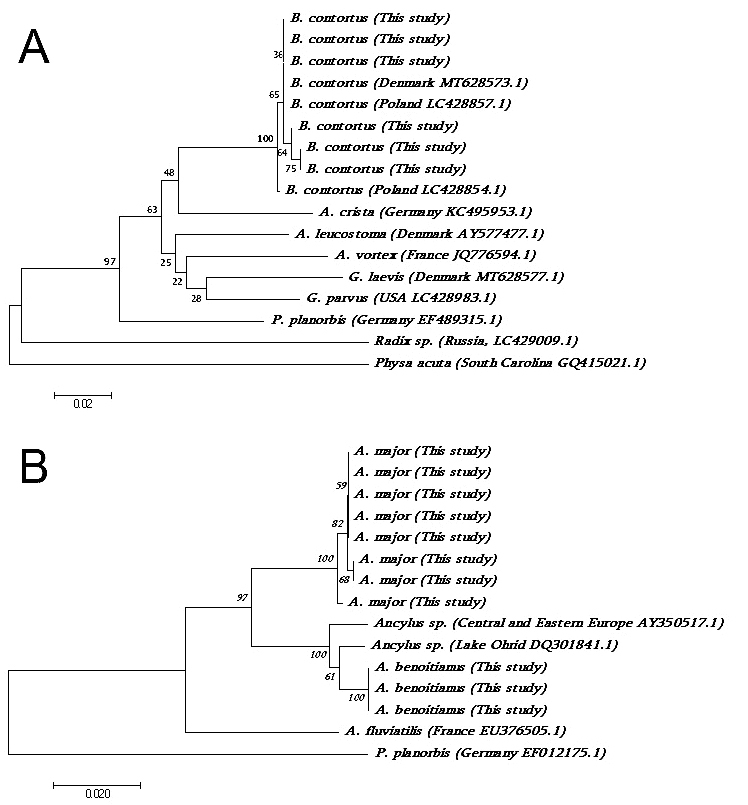
Neighbour Joining trees. **A.** *Bathyomphalus contortus* (16S rRNA mt gene fragment); **B.** *Ancylus* spp. (COI mt gene fragment). GenBank Accession numbers and sample origin places are indicated for downloaded sequences. Branch length (and scale-bar) resembles the nucleotide divergence (K2P distance) and the numbers at the node indicates bootstrap support after 100 permutations.

The specimens of *B. contortus* were collected in both western and eastern parts of Javakheti Highlands (Suppl. material 2). Shell morphological characters of *B. contortus* were typical, as reported elsewhere, for example, Glöer (2002), Welter-Schultes (2012). In particular, shells are thick-walled, small, discoidal, with yellowish to light – brown periostracum with 7 – 8 high and very narrow whorls, which are flattened on their lower sides. The shell surface is delicately striated. The umbilicus is very deep and the aperture is narrow and crescent-shaped. Maximum shell heights are up to 2 mm and widths up to 6 mm. The morphological identification was further supported by 16S rRNA sequences (400 pb) obtained from three specimens (GenBank accession numbers: MW694834-37; MW694843-44). Comparison of the 16S rRNA sequences which we obtained with GenBank data, showed 98-100% identity with published sequences and clustered with *B. contortus* samples from Northern Europe (e.g. Saito et al. 2018). The average divergence between Georgian and European specimens was 0.45%. Thus, despite the low bootstrap support of the *Bathyomphalus* clade on the NJ tree (Fig. 4), our samples can still be unambiguously identified as *B. contortus*.

**Figure 5.**
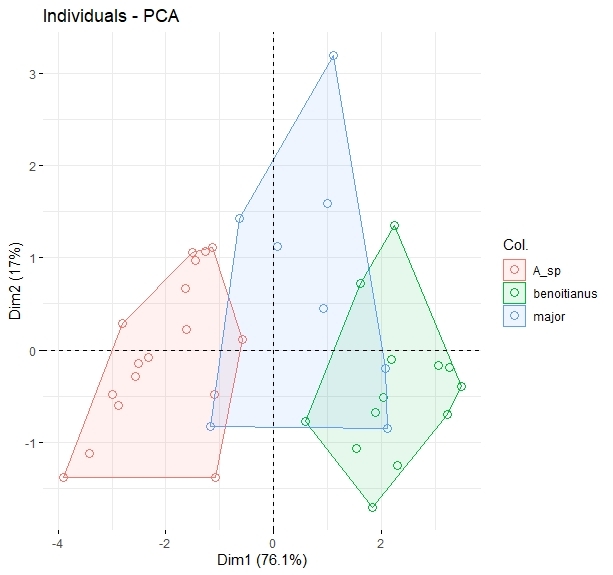
Arrangememnt of *Ancylus* specimens on ordination graphs after Principal Component Analyses of shell measurements (measurements are given in Suppl. material 1). First two components describe more than 93% of shell variation and the first component (mapped on X-axis) was able to successfully descriminate between the three putative species of *Ancylus* from Javakheti Highlands.

Amongst the collected material, we also detected three different morphotypes of planorbid genus *Ancylus*. From these morphotypes, two of them were morphologically identified as *A. major* (8 specimens, SH = 6 (0.85 sd), HW = 5.6 (0.53 sd), SH = 2.8 (0.49 sd)) and *A. benoitianus* (14 specimens, SH = 7 (0.56 sd), HW = 5.4 (0.34 sd), SH = 3.4 (0.29 sd)) according to shell characters provided by Soldatenko and Starobogatov (2004). However, a third form (*Ancylus* sp., 17 specimens, SH = 3.7 (0.72 sd), HW = 3 (0.57 sd), SH = 1.36 (0.38 sd)) could not be allocated to any previously-described *Ancylus* taxa known from the region. In particular, the characters distinguishing the shells of *Ancylus* sp. from the others are the overall small size and the smallest relative shell height, ever recorded from Eurasian representatives of *Ancylus* (Soldatenko and Starobogatov 2004). Indeed, the shell height/length ratio varies from 0.29-0.45 (with an average 0.36). In addition, the apex of *Ancylus* sp. is much more slightly curved and less developed compared to any other *Ancylus* taxa known to occur in the Caucasus. Multivariate ordination (PCA), based on the shell measurements, clearly separated the *Ancylus* sp. from the other congenerics (Fig. 5).

We were able to obtain COI DNA sequences (up to 560 bp) for only eight specimens of *A. major* and three specimens of *A. benoitianus* (GenBank Accession numbers: MW703500-09; MW680406). BLAST searches indicated 92-99% similarity with sequences of *Ancylus* taxa sampled from central and eastern Europe (Pfenninger et al. 2003, Albrecht et al. 2006, Cordellier and Pfenninger 2008). An NJ tree of our specimens and the others downloaded from GenBank revealed two well-supported clades (Fig. 4), one clade containing the specimens morphologically identified as *A. benoitianus* and the other to *A. major*. The divergence (measured as Kimura-2-parameter parameter distances) between the benoitianus/major clades is on average 5%, while within-clade distances do not exceed 1%. In addition, *A. benoitianus* has a lower divergence rate to the GenBank specimens from Central and Eastern Europe (1.5%) compared to *A. major* from Javakheti Highlands. Finally, divergence between all *Anclylus* taxa from the Javakheti region to *A. fluviatilis* from Europe (Cordellier & Pfenninger, 2008) exceeds 7%.

## Discussion

The Javakheti Highlands are one of the most important wetland-containing regions in the Caucasus. This importance was recognised in 1996 and again in 2011 by the establishment of the Ktsia-Tabatskuri and Javakheti protected area systems, respectively (http://apa.gov.ge/en/). In addition, the Javakheti Highlands were also recognised as one of the Important Bird Area sites by Birdlife International (BirdLife International 2021). However, the ‘importance’ of the Javakheti wetlands was recognised entirely based on their importance for bird species, not due to recognition of their intrinsic value as wetlands and most of the resident freshwater biodiversity is still very poorly studied. As an example, very little was known about freshwater molluscs of the Javakheti Highlands before our study. Only a few fragmentary and very old data exist, according to which, ten species (Table 2) were known from the region (Sadovskii 1933, Tskhomelidze et al. 1961, Kakauridze 1963). Within the present study, we were able to re-collect all the previously-known taxa and, in addition, 13 species (including three putative species of genus *Ancylus*) new for the region. Out of 13 new records, 10 species have either relatively narrow distribution ranges (e.g. *S. palustris*, *A. leucostoma*) or are rare (sporadically distributed), though widespread (e.g. *P. intermixtus*, *B. contortus*). This alone can explain the paucity of knowledge of freshwater mollusc diversity in the Region, compounded by the lack of research.

The planorbiid species *B. contortus* has never been reported from Georgia before. This species is known from Europe and northern Asia, also from neighbouring Armenia (Akramowski 1976, Mashkova et al. 2018). Thus the finding of *B. contortus* in Georgia is not very surprising and P. Glöer included Georgia in the distribution map of *B. contortus* in his book (Glöer 2019). Nevertheless, the distribution of *B. contortus* in Armenia and Turkey is restricted to waterbodies of continental mountain climate and the Javakheti Highlands is the part of this climate region. Consequently, we predict that the *B. contortus* is not widespread in Georgia, but rather is restricted to Javakheti Highlands and is probably found in Georgia only along the southern part of Kura River basin.

Another interesting finding concerns the *Ancylus* species-complex. According to Javelidze (1973), only *A. fluviatilis* was considered to be distributed in Georgia and within the whole South Caucasus, in general (Akramowski 1976). Subsequently, based on morphological revision, Soldatenko and Starobogatov (2004) distinguished five species that might occur in the Caucasus, but not *A. fluviatilis* (Vinarski and Kantor 2016). However, due to limitations of species-specific morphological and anatomical characters, the identity of *Ancylus* species remains doubtful (Soldatenko and Starobogatov 2004, Sitnikova et al. 2012), not only in the Caucasus Region, but the species level taxonomy of European *Ancylus* is still uncertain (Pfenninger et al. 2003, Soldatenko 2009, Albrecht et al. 2006). Our preliminary determination of *Ancylus* taxa, collected in Javakheti Highlands, indicates the existence of at least three different morphotypes. From those morphs, two of them were identified as *A. benoitianus* and *A. major*, based on shell morphological characters. Specimens of these two taxa were also clustered in clearly separated clades in the NJ tree of COI sequences. Unfortunately, no comparative material of either species exists in GenBank/BOLD data repositories and the confirmation of species-level taxonomic status remains unresolved. The third morphotype of *Ancylus* does not resemble any of the *Ancylus* species proposed by Soldatenko and Starobogatov (2004) for the Caucasus Region. Instead, the shell shape most closely resembles *A. subcircularis* Clessin 1882 which is reported from Europe. Since we did not obtain DNA sequences for this morphotype, the status of this taxon remains fully unresolved and needs additional study.

Most of the species collected during the present investigation were found in standing water or slowly flowing river reaches, with dense submersed vegetation. Only a small portion of species, such as *Ancylus* spp., *P. peregra* and *A. hypnorum* are found in rivers with moderately to fast flowing currents. Although all the Javakheti Region belongs to the Kura River basin, the western and eastern part of the Region harbours few uniques species. Particularly interesting is the variation of species spatial distribution in comparison between closely-related taxa. As an example, *R. auricularia* is widespread all over the Javakheti Highlands, while *Ampullaceana lagotis* is restricted to the western part of the Javakheti Highlands in the Paravani basin and *P. peregra* to the eastern part (Khrami River basin). Similar patterns are also evident for species of genus *Ancylus* and the family Sphaeridae (Suppl. material 1). This kind of regional distribution pattern might well be related to biogeographical history, but that needs further research. We did not find *Physella acuta* (Draparnaud, 1805) in the study area, although this species is probably the most widespread of all molluscs within Georgia and the Caucasus. We surmise that the relatively cold, continental climate of the Highlands might prevent the spread of this species into Javakheti, as well as intraregional translocations for other resident species.

## Conclusions

The species diversity and distribution of Javakheti Highlands can be currently regarded as moderately well studied. However, considering the diversity of water bodies in the Region, further study is needed to fine-tune the species’ spatial distributions. In addition, molluscs are one of the principal components of freshwater ecosystems and thus should be subject to ongoing biological monitoring, given the vulnerability of freshwater ecosystems, which are only partially protected in the Javakheti Highlands.

## Supplementary Material

2C8EBA95-D3F3-5180-8173-4DCFD78F2EE1 10.3897/BDJ.9.e66649.suppl1 Supplementary material 1 Shell measurements of 39 specimens of the genus *Ancylus*.
Data type Morphological Brief description Shell measurements of 39 specimens of the genus Ancylus from Javakheti Highlands are presented. Abbreviations stand for: shell length (SL), shell width (SW), shell height (SH), shortest distance between apex and apertural margin (Ra), shortest distance between highest point and apertural margin (Rh) and height of lower part of shell near apex (Hsp) File: oo_525133.xlsx https://binary.pensoft.net/file/525133 Kachlishvili, Nino

69B32428-3D35-56B4-8CE6-4B1B121B25E4 10.3897/BDJ.9.e66649.suppl2 Supplementary material 2 Distribution of freshwater mollusc species in Javakheti Highlands Data type Distribution maps Brief description Maps of geographic distribution of each separate species in Javakheti Highlands are provided. File: oo_536015.JPG https://binary.pensoft.net/file/536015 Bikashvili, Ani

## Figures and Tables

**Table 1.** Geographic names, coordinates, absolute elevation above sea level (alt) in metres and short description of sampling sites. The last column indicates the number of species collected in each of the site.

Site code	Location/Habitat	Lat./Long.	Alt. (m a.s.l.)	Short description	Species
1	Apnia, Unnamed spring	41.366103; 43.279093	1691	Spring rich in mosses	1
2	Didi Tba Lake	41.35226; 43.34106	1787	Heavily eutrophic lake	5
3	Azmana River	41.318338; 43.321854	1721	Rich in vegetation with silty bottom	2
4	Kodalistskali River	41.295087; 43.323328	1855	Densley vegetated water body	1
5	Vachiani Lake	41.358505; 43.444788	1740	Disturbed lake (intensive water uptake during dry period, waste water discharge)	3
6	Little Lake near Khanchali Lake	41.254969; 43.534045	1931	Heavily eutrophic lake	2
7	Khanchali Lake	41.244391; 43.560145	1931	Disturbed lake (antropogenic activities)	10
8	Swamp near Balka Kamenistaia River	41.240439; 43.575199	1946	Swamp	4
9	Bughdasheni River	41.213233; 43.662639	2047	Stony bottom with densely vegetated banks	3
10	Bughdasheni Lake	41.202408; 43.688346	2045	Stony, sandy, and muddy bottom, partly swampy	2
11	Gorelovka, Zagranichnaia River	41.21149; 43.69395	2049	Tributary of Bughdasheni Lake with dense vegetation	1
12	Madatapa Lake	41.176144; 43.765445	2116	Heavily eutrophic lake	5
13	Paravani River	41.293089; 43.728821	2015	Sandy and stony bottom, with aquatic vegetation	3
14	Saghamo Lake	41.29661; 43.73364	2008	Sandy, and silty bottom with patches of dense submersed vegetation	4
15	Unnamed Lake	41.308391; 43.596465	1861	Euthropic lake with dense submersed vegetation	1
16	Avchala Lake	41.341791; 43.69073	2059	Silty bottom with dense vegetation	3
17	Paravani Lake	41.426258; 43.78042	2079	Stony, sandy and silty bottom with patches of dense vegetation	7
18	Akhali Khulgumo, swamp on Paravani Lake edge	41.48183; 43.8305	2081	Swamp	3
19	Abuli Lake	41.385283; 43.617328	2188	Heavily eutrophic lake	1
20	Chelingoli Lake	41.431313; 43.571718	2007	Sandy - silty bottom with negligible amount of vegetation	1
21	Baraletistskali River	41.564869; 43.512769	1725	Stony and silty bottom with densely vegetated margins	4
22	Channel next to Baraletistskali River	41.580161; 43.532514	1775	Densely vegetated channel	1
23	Swamp on Ktsia-Tabatskuri Managed Reserve	41.667339; 43.504105	2396	Vegetation rich temporary water bodies	2
24	Tabatskuri Lake	41.642314; 43.595395	1996	Deep lake, wirh silty and rocky bottom.	2
25	Swamp next to Bortborti River	41.64409; 43.79429	1761	Swamp	1
26	Panishgioli Lake	41.657429; 43.839355	1744	Heavily eutrophic lake	1
27	Swamp next to Ozni River	41.63998; 43.845676	1693	Swamp	1
28	Bortborti River	41.617701; 43.884544	1545	Stony and silty bottom river, densely vegetated on its margins	2
29	Uzungioli Lake (Santa)	41.658457; 44.005944	1753	Well-vegetated lake with silty bottom	2
30	Chili - Chili River	41.641425; 44.109193	1527	Rich in vegetation with sandy and stony bottom	2
31	Bareti Lake	41.659933; 44.168195	1630	Well-vegetated lake	2
32	Egrichai River (Korsuchai)	41.653798; 44.250464	1622	Silty bottom lake with dense vegetation	5
33	Tba Lake	41.664958; 44.269593	1758	Heavily eutrophic lake	1
34	Zhamindzori River	41.549769; 43.88726	1920	Silty and stony bottom river	1
35	Kuredere River	41.5139; 44.063881	1535	Sandy and stony bottom river	1
36	Unnamed Lake	41.532142; 44.112832	1548	Well-vegetated marsh lake	1
37	Unnamed Lake	41.509579; 44.120701	1581	Vegetation rich lake	1
38	Chochiani River	41.495964; 44.076884	1505	Stony bottom river, densely vegetated on its margins	1
39	Patara Ordaklo Lake	41.221634; 44.105706	1848	Heavily eutrophic lake	2
40	Spring next to Patara Ordaklo Lake	41.226513; 44.114001	1780	Disturbed lake (Agricultural activities)	2
41	River near Lake Bashplemy	41.264941; 44.143227	1654	Rich in vegetation with sandy and pebbled bottom	8
42	Mamutliskhevi River	41.308803; 44.197366	1221	Stony bottom river, densely vegetated on its margins	2

**Table 2.** List of species recorded in Javakheti Highlands. Sampling sites are coded using the numbers according to Table 1. Species with asterisk are those previously known from the study area.

Species	Sampling sites	Paravani River Baisin	Khrami River Basin
Lymnaeidae			
*Lymnaea stagnalis	2,4,5,7,11,12,14,15,16,17,31,37	+	+
*Galba truncatula	1,3,7,23,32,41	+	+
*Stagnicola palustris*	2	+	-
*Ampullaceana lagotis	5,6,7	+	-
*Radix auricularia	7,9,10,12,13,14,17,22,24,29,31,33	+	+
*Peregriana peregra	40,41	-	+
Physidae			
*Aplexa hypnorum*	30,32,41	-	+
Planorbidae			
*Planorbis planorbis	5,7,8,12,17,18,26,27,28,38,41, 42	+	+
*Planorbis intermixtus*	17,21,41	+	+
*Armiger crista	2,7,12,16,18,36	+	+
*Anisus leucostoma*	17	+	-
*Anisus spirorbis*	2,7,12,29,32,39	+	+
*Bathyomphalus contortus*	7,16,30,40,41	+	+
*Ancylus major*	9,13,21	+	-
*Ancylus benoitianus*	28,35,42	-	+
*Ancylus* sp.	13,21	+	-
Sphaeriidae			
*Sphaerium corneum*	17,20,41	+	+
*Musculium lacustre*	2,6,7,8,10,18,19,39,41	+	+
*Pisidium casertana	3,8,23,24,25,32,34	+	+
*Pisidium subtruncata	14,21	+	-
*Pisidium nitidum*	9,14,17	+	-
*Pisidium obtusale*	8,32	+	+
