# Supplementary figures and images for: Species diversity and distribution of freshwater molluscs of Javakheti Highlands (Republic of Georgia)
Source: Biodivers Data J. 2021 Jun 7;9:e66649. doi: 10.3897/BDJ.9.e66649 (PMC8203596; doi:10.3897/BDJ.9.e66649)

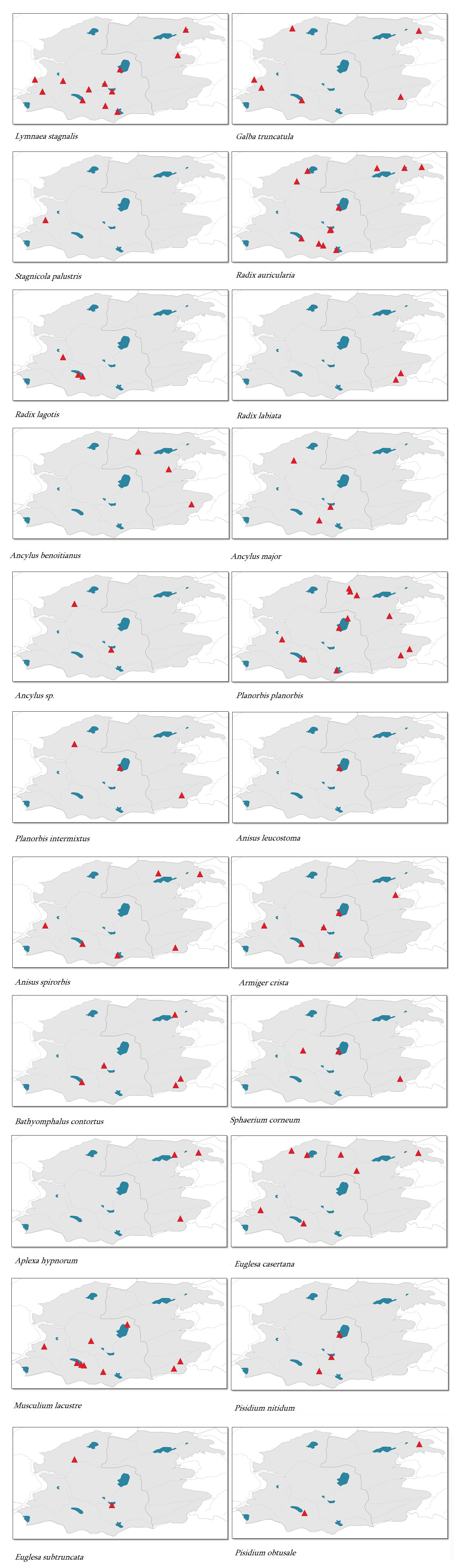

Supplement: Supplementary material 2 — Distribution of freshwater mollusc species in Javakheti Highlands [file bdj-09-e66649-s002.jpg]
